# Root System Architecture Plasticity of Bread Wheat in Response to Oxidative Burst under Extended Osmotic Stress

**DOI:** 10.3390/plants10050939

**Published:** 2021-05-08

**Authors:** Omar Azab, Abdullah Al-Doss, Thobayet Alshahrani, Salah El-Hendawy, Adel M. Zakri, Ahmed M. Abd-ElGawad

**Affiliations:** 1 Plant Production Department, College of Food and Agriculture Sciences, King Saud University, P.O. Box 2460, Riyadh 11451, Saudi Arabia; aaldoss@ksu.edu.sa (A.A.-D.); talshahrani@ksu.edu.sa (T.A.); mosalah@ksu.edu.sa (S.E.-H.); azakri@ksu.edu.sa (A.M.Z.); 2 Department of Agronomy, Faculty of Agriculture, Suez Canal University, Ismailia 41522, Egypt; 3 Department of Botany, Faculty of Science, Mansoura University, Mansoura 35516, Egypt

**Keywords:** oxidative stress, root system architecture, dichlorofluorescin diacetate, mitochondrial membrane stability

## Abstract

There is a demand for an increase in crop production because of the growing population, but water shortage hinders the expansion of wheat cultivation, one of the most important crops worldwide. Polyethylene glycol (PEG) was used to mimic drought stress due to its high osmotic potentials generated in plants subjected to it. This study aimed to determine the root system architecture (RSA) plasticity of eight bread wheat genotypes under osmotic stress in relation to the oxidative status and mitochondrial membrane potential of their root tips. Osmotic stress application resulted in differences in the RSA between the eight genotypes, where genotypes were divided into adapted genotypes that have non-significant decreased values in lateral roots number (LRN) and total root length (TRL), while non-adapted genotypes have a significant decrease in LRN, TRL, root volume (RV), and root surface area (SA). Accumulation of intracellular ROS formation in root tips and elongation zone was observed in the non-adapted genotypes due to PEG-induced oxidative stress. Mitochondrial membrane potential (∆Ψm) was measured for both stress and non-stress treatments in the eight genotypes as a biomarker for programmed cell death as a result of induced osmotic stress, in correlation with RSA traits. PEG treatment increased scavenging capacity of the genotypes from 1.4-fold in the sensitive genotype Gemmiza 7 to 14.3-fold in the adapted genotype Sakha 94. The adapted genotypes showed greater root trait values, ∆Ψm plasticity correlated with high scavenging capacity, and less ROS accumulation in the root tissue, while the non-adapted genotypes showed little scavenging capacity in both treatments, accompanied by mitochondrial membrane permeability, suggesting mitochondrial dysfunction as a result of oxidative stress.

## 1. Introduction

Increasing the world wheat production by 2050 by 1.6% per year, when the population would be expanding to 9 billion, is facing a lot of obstacles because of climate change [1]. There is ample evidence that the bread wheat productivity in arid regions is influenced by extended drought, causing a high reduction in grain yield [2,3]. Plants unable to escape from unfavorable conditions due to being sessile must be able to adapt to environmental challenges. During their evolution, plants evolved tremendous capabilities to sense alterations around themselves and rapidly respond by changing their growth directions [4]. Root system architecture (RSA) traits play a major role in plant growth and development due to their importance as an anchor in the soil substrate and for moisture and nutrients mining [5,6]. Deeper root system genotypes can access deep soil profile more than the shallow ones, resulting in a cooler canopy and higher grain yield performance under the conditions of normal, moderate, and severe drought stresses [7]. The leaf area and shoot biomass were affected in small root system genotypes in the early growth stages as a result of drought stress, where better performance was observed in high vigor-rooted genotypes [8]. It was suggested that maintenance of lateral root formation in rice plants under osmotic condition was correlated with strong shoot systems and high root biomass [9]. Selection for RSA plasticity under water stress conditions could be a point of interest for plant breeding programs for better water consumption and adaptation to abiotic stresses [10], which became an image-based high-throughput phenotypic technique, where a high set of genotypes can be evaluated in less time [11,12].

Plant roots are functionally characterized by the presence of three regions: a meristematic zone where cells exhibit a high rate of cell division [13], an elongation zone where cells elongate and begin to differentiate [14], and a maturation zone where root hair and lateral root formation and development take place [15]. There is abundant evidence that the presence of a reactive oxygen species (ROS) in the meristematic and elongation zones can act as signaling molecules, serving a functional role similar to hormones [16]. After osmotic stress treatment was induced by (PEG) 6000, the various increased synthesized metabolites maintained important functions such as energy production and antioxidant defense in rice seedlings [17]. In addition, the downstream products of ROS play a crucial role in modulating the auxin signaling pathways [18]; they are critical second messengers involved in drought stress in plants [19,20]. ROS are potentially harmful since they are regarded as a key factor in cellular components, macromolecules, and DNA damage, resulting in irreparable metabolic dysfunction [21,22]. Enzymatic activity of superoxide dismutase (SOD) in wheat plants resulted in drought tolerant primary root elongation under osmotic stress [23]. It acts as signaling molecules for controlling plant programmed cell death (PCD) [24]. Nair and Chung [25] found that increased ROS generation and lipid peroxidation resulted in reduced root growth in chickpea. Gui et al. [26] found that indole-3-acetic acid (IAA) production in rice was positively correlated with the antioxidant enzymes’ activity. Increased ROS generation reduced the root length and biomass of *Brassica juncea* [27]. Majumdar et al. [28] indicated that short-term exposure of 15-day-old kidney bean seedlings’ roots to cerium oxide nanoparticles activated peroxidase, which resulted in scavenging processes in the ROS production and combated the oxidative stress.

In a recent study, the mitochondria are considered a cross-point in PCD through ROS signaling pathways [29]. Although ROS are not only produced from the mitochondria, the mitochondrial membrane is still the primary target of ROS-induced damages [30]. Under stress conditions, the mitochondrial membrane potential (∆Ψm) and ROS production rate are increased in the mitochondria [31]. The amplification of ROS production lies in Complex I and Complex III in the electron transport chain (ETC) to regulate the ROS concentrations in the whole cell [32]. One of the ROS targets is the mitochondrial membrane lipids, which can lead to PCD through mitochondrial dysfunction [16].

This study focused on the ability of the RSA of different bread wheat genotypes to adapt to extended osmotic stress, and the relationship between osmotic stress-induced ROS overproduction in root tips and ∆Ψm as a source of adenosine triphosphate (ATP) to produce more adapted roots. RSA plasticity was measured using the WinRHIZO software [33]. This article aimed to reveal how osmotic stress modulated post-embryonic primary roots, using the specific dye rhodamine (Rh123) as a biomarker for programmed cell death in the non-adapted genotypes under osmotic stress, and the potential of redox generation due to the enzymatic capacity of the cell to reduce the accumulation of generated cellular ROS, that was tracked using 2′,7′-dichlorofluorescin diacetate (DCF-DA).

## 2. Materials and Methods

### 2.1. Plant Materials and Experimental Design

In this study, eight bread wheat genotypes collected from different geographical regions were investigated. These genotypes were selected based on their differences in RSA traits under drought conditions (Table S1). The seeds of the eight genotypes were surface-sterilized with 0.15% sodium hypochlorite for 20 min and washed twice with distilled and sterilized water. The seeds were germinated in Petri plates for four days, where the seedling radicle length reached around 1.5 cm. Eight seedlings of each genotype were transplanted into a magenta box as an experimental unit filled with autoclaved vermiculite. The magenta boxes were incubated in the growth chamber, adjusted with a light cycle of 14 h light and 10 h dark, and day/night temperatures of 21–23/16–18 °C, for 14 days. The experiment consisted of two treatments; each treatment contained eight genotypes with three replicates. A control treatment with only half-strength Murashige and Skoog basal medium and stress treatment with 10% polyethylene glycol (PEG 6000, SigmaAldrich Chemie, Steinheim, Germany), which was approximately equal to −0.45 MPa osmotic potential [34], were performed. The plants were inspected daily to check for osmotic stress symptoms, and the experiment was terminated 14 days after treatment (DAT).

### 2.2. RSA Trait Measurements

**Figure 1:**
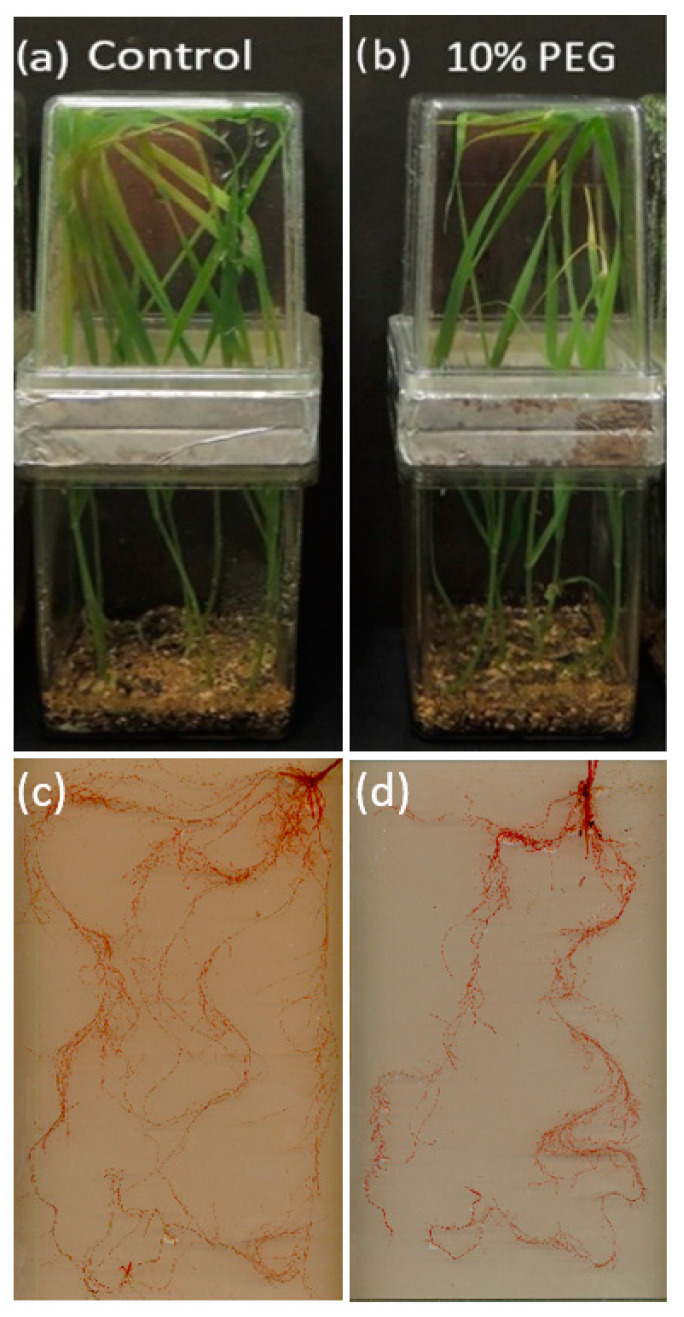
Wheat plants grew in magenta boxes. (**a**) control treatment, (**b**) 10% PEG 6000 (osmotic stress), (**c**,**d**) selected stained root for photo scanning and analyzing of control and PEG treatment, respectively.

Three replicates of roots from each genotype were prepared by extracting the plants from the magenta boxes and washing off the vermiculite. Then, the roots were stained with toluidine red for approximately 8 h before scanning (Figure 1, Figure S1) and scanned using a flatbed hp scanner (Scanjet, G2410, 1200 dpi); the photos were analyzed using WinRHIZO software (V5.0, Regent Instruments, Quebec, Canada) [33]. The selected RSA functional traits, including the total root length (TRL), lateral root number (LRN), root volume (RV), and surface area (SA), were determined.

The relative drought effect (RDE) of the traits was calculated using the following equation:

$$\mathrm{RDE}=\frac{\left(\mathrm{average}\;\mathrm{of}\;\mathrm{trait}\;\mathrm{under}\;\mathrm{control}\;\mathrm{condition})-(\mathrm{average}\;\mathrm{of}\;\mathrm{trait}\;\mathrm{under}\;\mathrm{stress}\;\mathrm{condition}\right)}{\mathrm{average}\;\mathrm{of}\;\mathrm{the}\;\mathrm{trait}\;\mathrm{under}\;\mathrm{control}\;\mathrm{condition}}\times 100$$

### 2.3. Measurement of ROS in Root Tips

The generated ROS in root tips due to osmotic stress were measured according to the method of Duan et al. [35]. Fourteen DAT seedlings from the control and 10% PEG treatment were incubated in 0.25 µM DCFH-DA in 1× PBS buffer for 15 min and washed twice with PBS buffer; finally, these were imaged using a fluorescence microscope (Nikon Eclipse 80i, Tokyo, Japan) at excitation and emission wavelengths of 485 and 530 nm, respectively. To obtain an accurate result, the experiment was repeated three times.

### 2.4. Visualization of Mitochondrial Membrane Potential (∆Ψm)

The fluorescence intensity of mitochondria-specific dye, rhodamine (Rh123), was monitored to track the ∆Ψm changes in root tips as described by Saquib et al. [36]. The root tips from the control and 10% PEG treatment were stained with 20 μM mL^−1^ of Rh123 for 30 min at 37 °C in the dark, and visualization was done through a fluorescence microscope (Nikon Eclipse 80i, Tokyo, Japan) at an excitation wavelength of 520 nm and an emission wavelength of 590 nm.

### 2.5. Antioxidant Activity Determination

The antioxidant activity of the root extract was determined according to its ability to reduce the stable radicle DPPH (Sigma–Aldrich, Merck KGaA, Darmstadt, Germany), following the method of Sharma and Bhat [37]. A reaction mixture of 2 mL of DPPH (0.15 mM) and an equal amount of root crude extract (either stressed or non-stressed) with various concentrations (125, 250, 500, 750, and 1000 mg mL^−1^) in methanol were prepared in glass tubes. The tubes were vigorously shacked and incubated in a dark condition at room temperature (25 °C). After 30 min, the absorbance was measured by spectrophotometer (Ultrospec 2100 pro UV/Visible spectrophotometer, Amercham Biosciences, Cambridge, UK) at 517 nm. A control was prepared using methanol and underwent the same treatments. The antioxidant scavenging activity was calculated in percentage using the following equation:

Radical scavenging activity (%) = [1 − (Absorbance _treatment_/Absorbance _control_)] × 100

The IC_50_ was calculated as the value expresses the amount of sample necessary to decrease the absorbance of DPPH by 50%.

### 2.6. Statistical Analysis

The experiment was designed with three replications for each genotype. The data of RSA and antioxidant activity were analyzed with two-way ANOVA tests using COSTAT Version 3.03 (software, Berkeley, CA, USA). The mean values were compared using Duncan’s multiple range test at a probability level of 0.05.

## 3. Results

### 3.1. Phenotypic Variation in RSA

**Figure 2:**
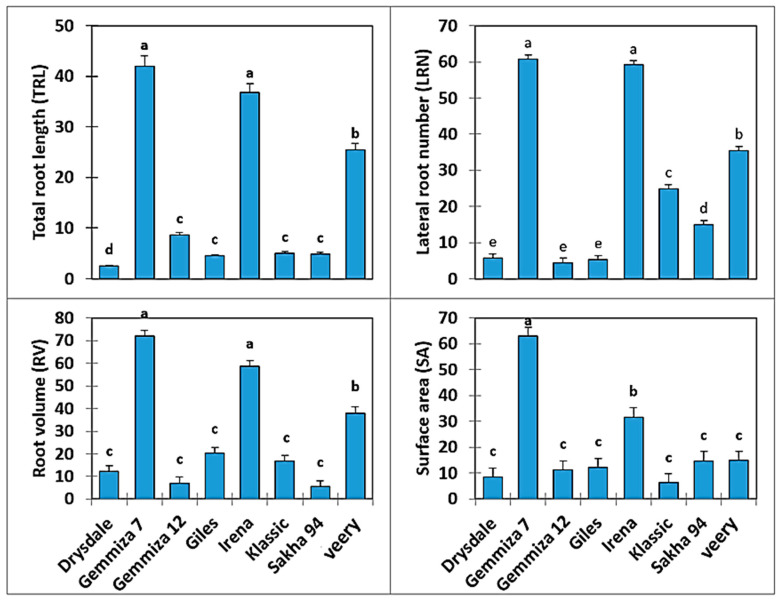
Relative drought effect of RSA traits for eight bread wheat genotypes at 14 DAT. Different letters within each parameter means significant difference at probability level of 0.05.

The ANOVA tests showed that water stress caused significant differences in the RSA traits of the studied genotypes (Figure 2). All measured root traits were reduced under stress conditions relative to the control. However, Gemmiza 7, Irena, and Veery genotypes showed the highest relative decrease in values for all RSA traits, and the opposite was true for Drysdale, Giles, Sakha 94, and Gemmiza 12. The Klassic genotype attained less relative decrease in values for root SA and LRN, but higher value for RV and TRL (Figure 2).

### 3.2. Antioxidant Activities

**Figure 3:**
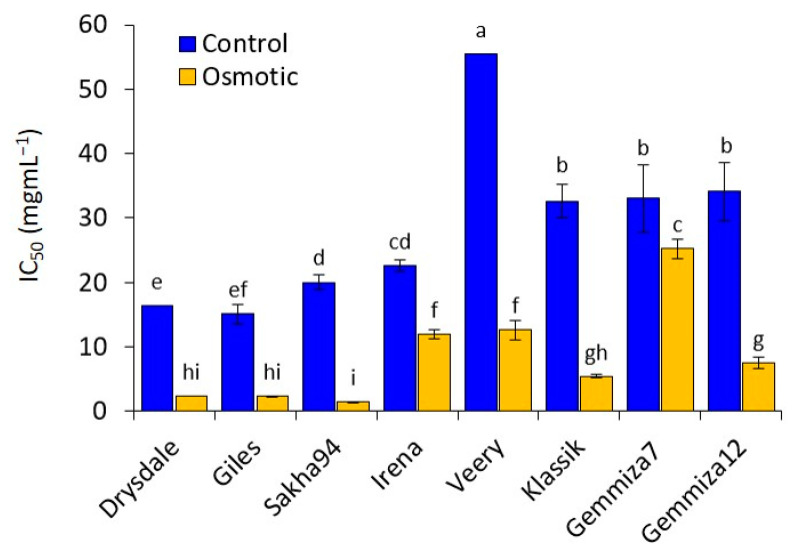
Calculated IC_50_ for root extracts 14 DAT, demonstrating a genotype × treatment interaction (*p* = 0.0001). Data points within genotypes and treatment having the same letter are not significantly different at the 95% level of confidence.

Our results indicated that all the genotypes’ root extracts (Table S2) showed an increase in antioxidant capacity under osmotic treatment compared to the control. Based on the DPPH assay, Drysdale, Giles, Sakha 94, Gemmiza 12, and Klassic genotypes showed lower IC_50_ percentages under osmotic stress treatment (Figure 3) than the control. Gemmiza 7, Irena, and Veery showed much higher IC_50_ percentage values under the osmotic stress treatment (Figure 3). In addition, the adapted genotypes such as Sakha 94, Drysdale, Giles, Gemmiza 12, and Klassic had high scavenging percentages within all genotypes under the osmotic treatment, while Gemmiza 7, Irena, and Veery were considered the sensitive genotypes, with low scavenging percentages under osmotic treatment.

### 3.3. Effect of PEG on Intracellular ROS Generation and Mitochondrial Activity

**Figure 4:**
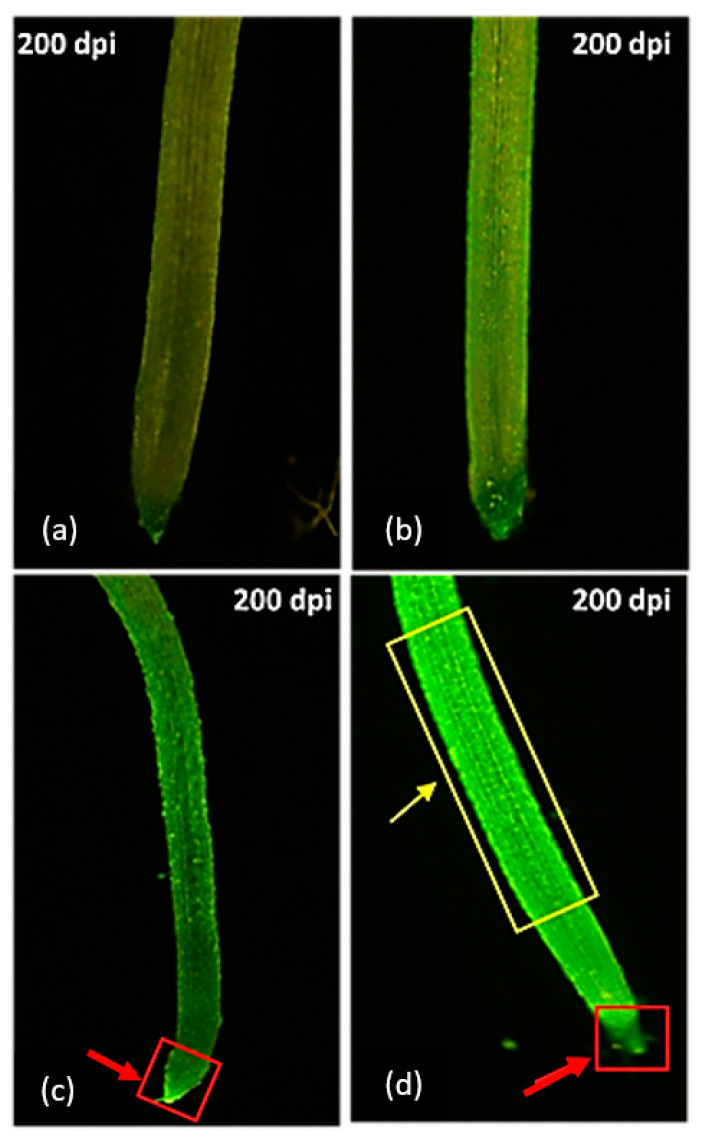
ROS generation in eight bread wheat seedling roots at 14 DAT. (**a**) Control for adapted genotypes; (**b**) control for non-adapted genotypes; (**c**) osmotic treatment for adapted genotypes; (**d**) osmotic treatment for non-adapted genotypes. In sensitive genotypes’ root tip, the areas of elongation and differentiation exhibiting ROS localization are marked with red- and yellow-colored quadrangles, respectively.

Compared to the control treatment, 10% PEG 6000 induced an increase in DCF fluorescence in all genotypes (Figure S2). A much lesser DCF fluorescence enhancement was observed in the root tip (Figure 4c) of adapted genotypes, while a sharp increase in fluorescence was observed in the root tip and elongation area of non-adapted genotypes (Figure 4d).

**Figure 5:**
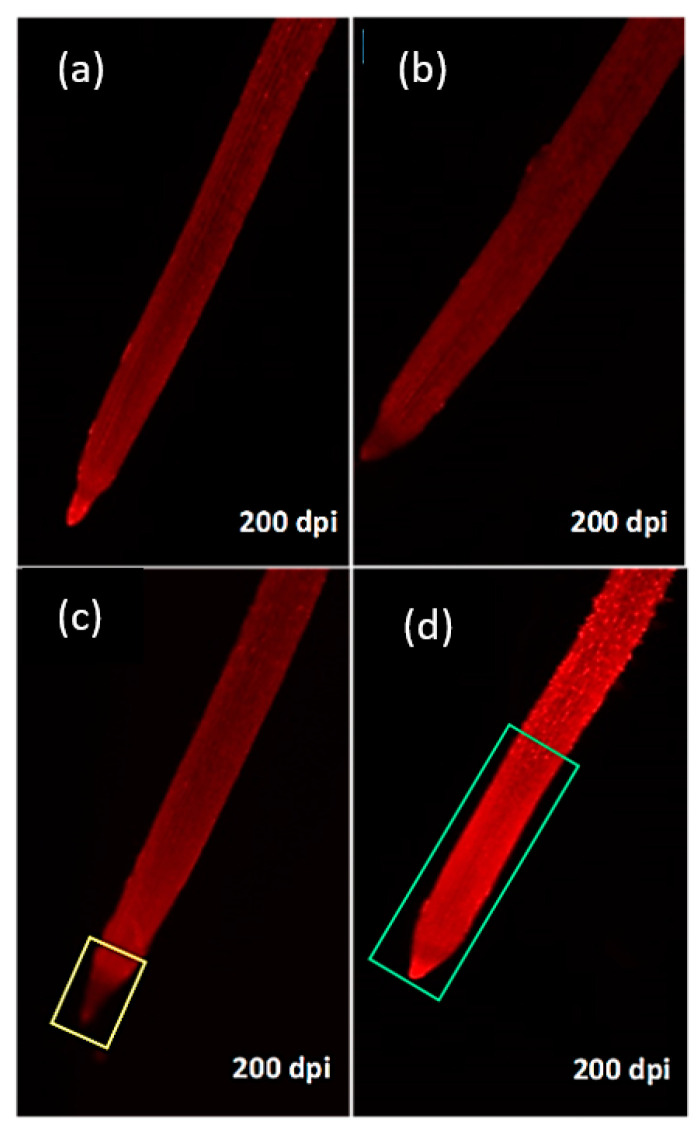
Rh123-stained root tips showing normal bright fluorescence in control treatment in the adapted genotype (**a**) and non-adapted genotype (**b**). Root tip meristem fluorescence decline at 10% PEG treatment (**c**) marked with yellow-colored quadrangle, and a hyperpolarization status at 10% PEG treatment in sensitive genotypes (**d**) marked with green-colored quadrangle.

Visualizing the changes in ∆Ψm in PEG-treated genotypes, the adapted genotypes’ root tip meristems exhibited a lesser reduction in Rh123 fluorescence (Figure 5c), while a discernibly high fluorescence intensity under the osmotic stress treatment was observed in non-adapted genotypes, possibly as a result of diffusion into the cytoplasm compared to the control (Figure 5d). In general, osmotic stress-adapted genotypes Drysdale, Giles, Sakha 94, Klassik, and Gemmiza 12 showed high RSA plasticity and maintained a normal fluorescence in the elongation area, with a lesser decrease in the meristematic area. Non-adapted genotypes Irena, Gemmiza 7, and Veery had low RSA plasticity and high fluorescence intensity, especially in the elongation area (Figure S3).

## 4. Discussion

Modulation of RSA and the degree of RSA plasticity affect the above-ground part’s growth and development, where the different RSA components maintain better water and nutrient uptake [38]. RV was one of the most important traits for water absorption [39], where better shoot development was correlated with a vigorous root system and LRN in early growth stages [40]. However, water-limiting conditions resulted in a decrease in root system traits, such as dry matter, length, LRN, and the diameter of nodal roots in drought-sensitive genotypes [41,42]. Genotypes with large root length had better adaptation to drought conditions due to their ability to access deep soil profiles [43,44]. Our results showed that Drysdale, Giles, Sakha 94, and Gemmiza 12 showed a more extensive and vigorous root system under the control and osmotic treatments (Figure 2). The morphological plasticity of RSA is considered the most important difference between the sensitive and tolerant genotypes. In the present study, Gemmiza 7 showed the highest reduction in TRL (40%), LRN (60%), RV (55%), and SA (60%), followed by Irena and Veery, which had the same performance for RSA traits, except for root SA.

The well-developed and vigorous root systems led to vigorous shoot systems [40]. Thus, adapted roots lead to vigorous biomass and, in turn, high yield. Figueroa-Bustos et al. [8] reported that small root system genotypes resulted in decreased leaf area and shoot biomass under drought stress, while deep-rooted genotypes had high recovery. In this study, the genotypes Drysdale, Giles, Sakha 94, and Gemmiza 12 had the most adapted RSA under osmotic stress. Drought-tolerant rice seedlings showed high root biomass through maintained increased LR development [9]. Adapted genotypes showed decreases in TRL and LRN by 3–8% and 3–15% under osmotic stress, while non-adapted genotypes showed decreases of 25–42% and 25–60%, respectively, when compared with the control treatment. This led to the decrease in the RV and SA of non-adapted genotypes. These differences suggest that the roots of adapted genotypes grew more vigorously than the non-adapted ones due to a specific physiological function.

Since RSA is closely related to water stress tolerance [43,44,45], it is essential to determine whether the osmotic stress-induced ROS in seminal root tips might provide a protective mechanism for RSA maintenance as a response to continuous severe osmotic stress or stimulate PCD, leading to a decreased RSA. PEG oxidative stress capability was examined through the visualization of ROS generated by staining the bread wheat seedlings’ seminal roots with DCF-DA staining (Figure 4a–d). DCF fluorescence was considered a marker for oxidative stress and overall oxidative status [46].

Jacomini et al. [34] hypothesize that PEG can accumulate in extracellular spaces, inducing cellular but not tissue dehydration. The development of RSA traits is controlled by many gene networks, where polar auxin transport carriers in the plasma membranes play a role in auxin transport and homeostasis, which are involved in lateral root formation [47], root development, embryogenesis, and organogenesis [48,49,50]. ROS are also involved in various processes, such as root gravitropism [51]. The formation of O_2_ ^•−^ and H_2_O_2_ functions as signaling molecules for cell elongation, differentiation, and lateral root formation [16,52]. ROS play a dual role, causing damage and signaling to induce defense mechanisms [53,54]. When ROS reach damaging levels, plants trigger the antioxidants as a defense strategy [55], where the activity of antioxidant enzymes is observed in the primary roots of tolerant wheat seedling under osmotic stress [23].

In accordance with previous results, normal DCF fluorescence was observed in the control treatment and adapted genotypes under stress, while non-adapted genotypes under stress showed high fluorescence intensity, especially in the elongation area, which is the region responsible for differentiation and lateral root formation. These results are consistent with the quantitative DPPH analysis of the root extract of all genotypes as a response for the total antioxidant capacity (Table S2). IC_50_ reflects the amount of tissue needed to decrease the absorbance of DPPH by 50%. The total antioxidant capacity of root extracts showed high IC_50_ values, which reflects a low scavenging potential for the overproduction of ROS that could cause damage to the root cell components. In general, the total antioxidant capacity for all genotypes increased in osmotic treatment compared to the control. However, adapted genotypes showed low IC_50_ percentages, while non-adapted genotypes showed high IC_50_ percentages, suggesting a correlation between RSA adaptability and total antioxidant scavenging capability.

Liszkay et al. [56] suggested that the overproduction of O_2_ ^•−^, H_2_O_2_, and ^•^OH can be demonstrated in the primary roots of maize (*Zea mays*), causing wall loosening and inhibition of elongation. Chen and Fluhr [57] reported that the high-molecular weight PEG treatment on 7-day-old *Arabidopsis* seedling roots resulted in the production of singlet oxygen, followed by cell death. Thus, the accumulation of ROS in the osmotic treatment might play a critical role in inducing cellular damage, leading to apoptotic-like PCD. This could be the reason for the low TRL and LRN in the sensitive genotypes.

Tsukagoshi [16] suggested that high-molecular mass molecules, such as mitochondrial DNA or membrane lipids, are the preferred targets for ROS; mitochondrial dysfunction takes place due to the high conductance of mitochondrial permeability transition (MPT). In this study, the specific dye of mitochondrial membrane, Rh123, was used to analyze the ∆Ψm of adapted and non-adapted roots to reaffirm the ROS-mediated membrane damage.

The impairment of the mitochondrial ETC resulted in elevated intracellular oxidative stress, which causes mitochondrial damage [58,59], leading to mitochondrial dysfunction. The increased fluorescence intensity in non-adapted genotypes is quite intriguing, as the lesser reduction in fluorescence intensity in adapted genotypes could be due to the disruption of the inner membrane permeability, resulting in the dissipation of ∆Ψm. On the other hand, the increased fluorescence in sensitive genotypes could be related to the change in the property of mitochondrial size in response to the changes in ∆Ψm. Morphological transformation could alter the intensity of Rh123 fluorescence [60]. The Rh123 leakage from the mitochondrial membrane into the cytoplasm is caused by membrane damage [46,61]. Thus, the hyperpolarization of the Rh123 of sensitive genotypes in the osmotic stress treatment could be due to the stain released to cytosolic components. Hence, it was concluded that the antioxidant defense of mitochondria in wheat roots under severe water stress was more efficient than the cellular defense, resulting in less membrane damage and better retention of root-relative water content [62]. The analysis of transcripts showed the specific response of wheat roots to drought, including antioxidative enzymes, where two mitogen-activated protein kinases, as well as SOD, CAT, glutathione reductase, and flavin-containing monooxygenase, play an essential role in RSA adaptation to drought conditions [63]. In this study, a high scavenging percentage was observed in adapted genotypes under severe extended osmotic stress treatment, which showed low DCF-DA fluorescence intensity (Table S2), while non-adapted genotypes showed high ROS accumulation, with decreased RSA traits and lower scavenging percentages under the osmotic stress treatment.

## 5. Conclusions

The continuous severe osmotic stress of PEG 6000 to wheat roots resulted in significant repression of RSA traits, causing oxidative imbalance marked with antioxidant enzyme enhancement as a manifestation of proposed PCD. The results indicate the ability of ROS to trigger the dysfunction of mitochondrial permeability. Thus, continuous severe osmotic stress is proposed to stimulate PCD via LRN depression, which is considered an important component of SA and RV. The adapted genotypes showed low ROS accumulation and lesser ∆Ψm, which reflects the importance of antioxidant capacity to modulate the ROS overproduction, which could be the main reason for cell membrane stabilization. Future studies on auxin transport genes profiling, transcription factors, and antioxidant enzymes are recommended to determine the possible mechanism(s) for ROS scavenging in root cells.

## Data Availability

The data presented in this study are available in the article.

## Supplementary Materials

The following are available online at https://www.mdpi.com/article/10.3390/plants10050939/s1, Table S1: Origin and pedigree of eight genotypes represent different geographical regions, Table S2: Scavenging activity of different root extract concentration of various wheat genotypes under control and osmotic treatments, Figure S1. Eight bread wheat genotypes were grown in magenta boxes. Figure S2: DCF-stained root tips of each genotype under both treatments, Figure S3: Rh123-stained root tips of each genotype under both treatments.
