# Supplementary material for: Root System Architecture Plasticity of Bread Wheat in Response to Oxidative Burst under Extended Osmotic Stress
Source: Plants (Basel). 2021 May 8;10(5):939. doi: 10.3390/plants10050939 (PMC8151492; doi:10.3390/plants10050939)
Supplement: Supplementary file 1 [file plants-10-00939-s001.zip › plants-1204603-supplementary.pdf]

plants

MDPI

Supplementary Materials

Table S1. Origin and pedigree of eight genotypes represent different geographical regions

1	Drysdale	Australia	Hartog x 3 / Quarrion
2	Giles	Australia	Jauz/Vulian
3	Irena	International Maize and Wheat Improvement Center (CIMMYT)	BUCKBUCK/FLICKER//MYNA/VULTURE
4	Sakha 94	Egypt, Agricultural Research Center	Opata/Rayon//Kauz
5	Verry	USA	KAVKAZ/BUHO//KALYANSONA/BLUEBIRD
6	Klassic	USA	KLEIN-RENDIDOR/2*SONORA-64//INIA-66/3/CIANO-67/4/YECORA-70
7	Gemmiza 7	Egypt, Agricultural Research Center	CMH 74A.360 / SX // SERI 8213 / AGENT CGM4611-2GM-3GM-1GM-0GM
8	Gemmiza12	Egypt, Agricultural Research Center	OTUS/3/SARA/THB//VEE

Table S2. Scavenging activity of different root extract concentrations of various wheat genotypes under control and osmotic treatments. The values are average (*n* = *3*) ± standard deviation

**Treatment**	Concentration (g mL-1)				
1000	750	500	250	125	
**Control**					
Drysdale	3.840	3.522	2.658	2.249	0.818
	±0.085	±0.179	±0.056	±0.111	±0.147
Giles	3.976	2.886	2.249	1.659	0.841
	±0.232	±0.116	±0.096	±0.085	±0.179
Sakha94	3.704	3.272	2.954	2.068	1.500
	±0.085	±0.056	±0.085	±0.116	±0.167
Irena	2.363	2.000	1.659	0.818	0.432
	±0.085	±0.064	±0.116	±0.167	±0.085
Veery	0.886	0.477	0.318	0.182	0.078
	±0.056	±0.056	±0.032	±0.032	±0.008
Klassic	1.704	1.295	0.863	0.523	0.341
	±0.056	±0.056	±0.140	±0.032	±0.056
Gemmiza7	1.659	1.318	1.000	0.523	0.318
	±0.085	±0.085	±0.179	±0.085	±0.085
Gemmiza12	2.045	1.795	1.454	1.113	0.682
	±0.111	±0.032	±0.085	±0.032	±0.096
**Osmotic**					
Drysdale	30.479	26.324	23.379	19.909	16.644
	±0.148	±0.196	±0.141	±0.085	±0.148
Giles	31.347	24.429	20.411	20.114	16.301
	±0.318	±0.613	±0.148	±0.171	±0.168
Sakha94	39.132	34.612	29.543	24.817	15.799
	±0.141	±0.196	±0.196	±0.196	±3.569
Irena	5.959	3.790	3.151	2.374	2.146
	±0.112	±0.171	±0.202	±0.085	±0.085
Veery	5.685	4.247	3.881	3.037	1.826
	±0.148	±0.202	±0.141	±0.171	±0.180
Klassic	10.822	7.055	5.000	3.196	2.740
	±0.437	±0.148	±0.202	±0.141	±0.056
Gemmiza7	2.329	2.009	1.438	0.982	0.639
	±0.244	±0.116	±0.112	±0.196	±0.085
Gemmiza12	20.137	19.155	17.740	16.849	16.050
	±0.202	±0.085	±0.112	±0.256	±0.233

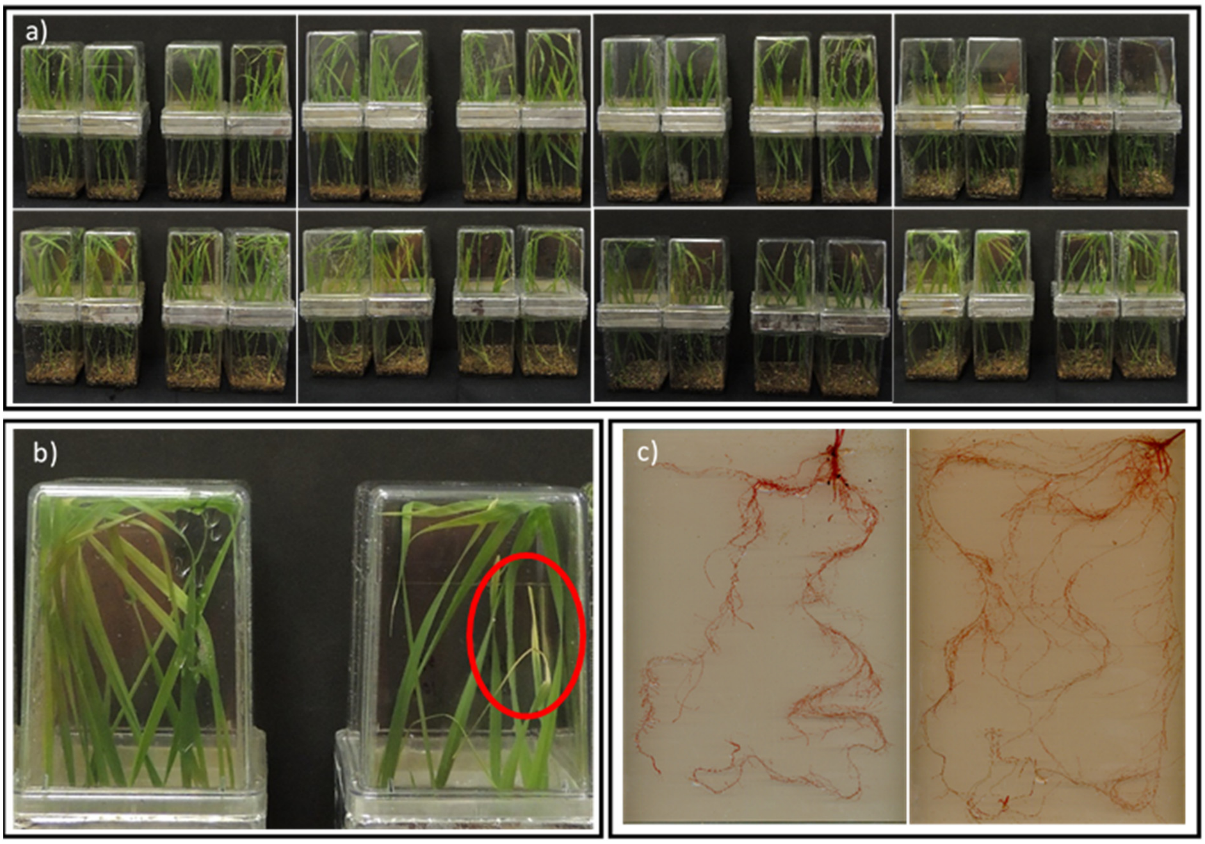

Figure S1. Eight bread wheat genotypes were grown in magenta boxes. **a)** Selected two magenta boxes from three replicates under both treatments, where the control treatment to the left and the osmotic treatment to the right. **b)** indicating stress signs inside the red circle after 14 days after treatment, where control treatment to the left and the 10% PEG treatment on the right. **c)** two selected genotypes roots, where adapted genotype to the right and the non-adapted genotype to the left.

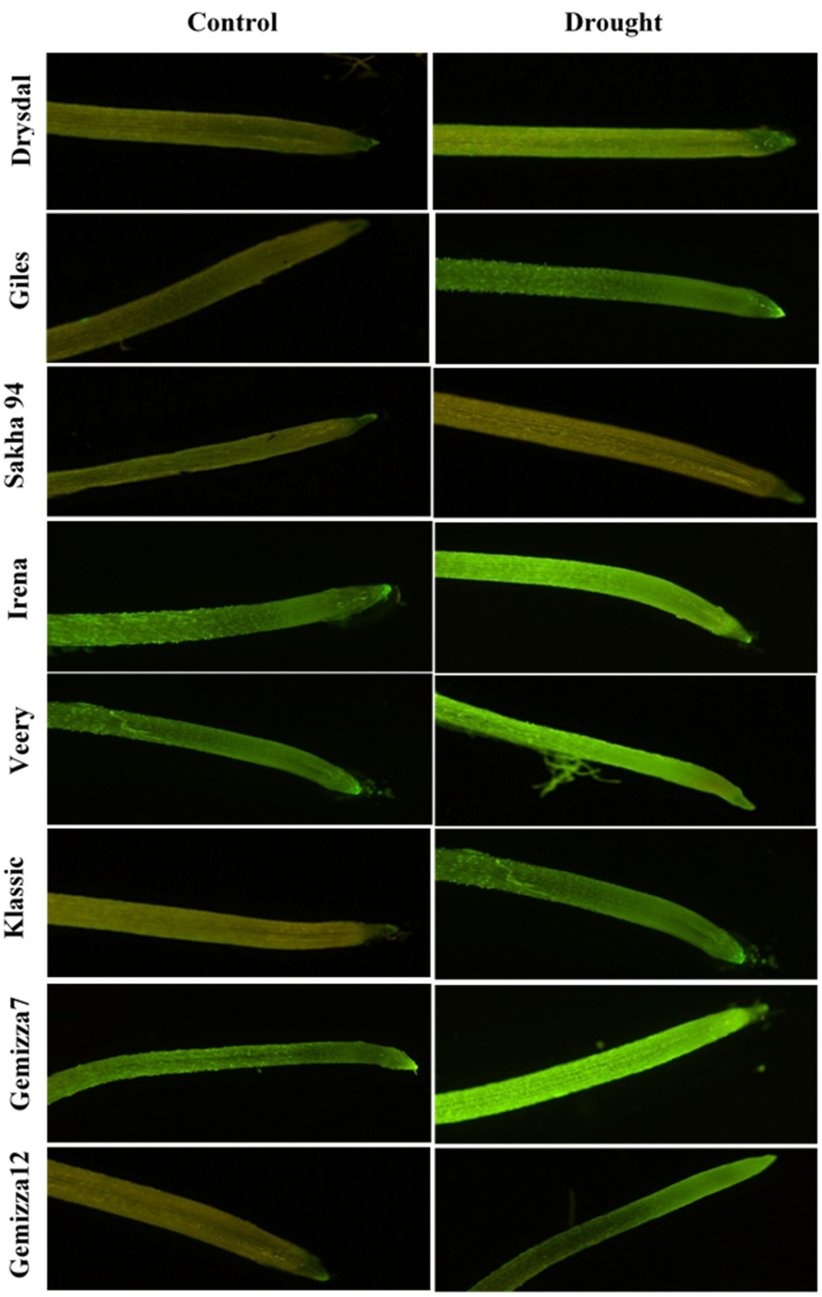

Figure S2. DCF-stained root tips of each genotype under both treatments, control treatment to the left and the 10% PEG treatment on the right indicating ROS accumulation due to treatment.

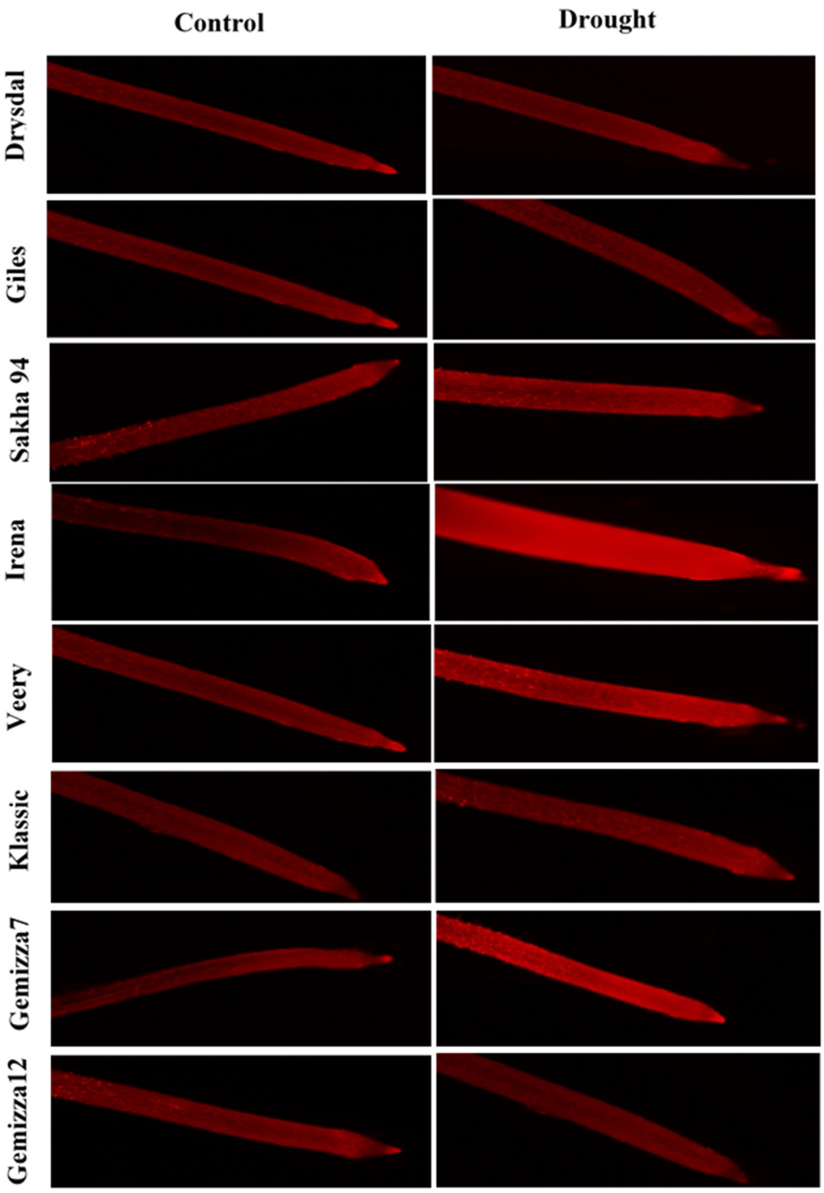

Figure S3. Rh123-stained root tips of each genotype under both treatments, control treatment to the left and the 10% PEG treatment on the right.
